# The effect of aging on context use and reliance on context in speech: A behavioral experiment with Repeat–Recall Test

**DOI:** 10.3389/fnagi.2022.924193

**Published:** 2022-07-22

**Authors:** Jiayu Sun, Zhikai Zhang, Baoxuan Sun, Haotian Liu, Chaogang Wei, Yuhe Liu

**Affiliations:** ^1^Department of Otolaryngology Head and Neck Surgery, Peking University First Hospital, Beijing, China; ^2^Department of Otolaryngology Head and Neck Surgery, Beijing Chao-Yang Hospital, Capital Medical University, Beijing, China; ^3^Widex Hearing Aid (Shanghai) Co., Ltd, Shanghai, China; ^4^Department of Otolaryngology Head and Neck Surgery, West China Hospital of Sichuan University, Chengdu, China; ^5^Department of Otolaryngology Head and Neck Surgery, Beijing Friendship Hospital, Capital Medical University, Beijing, China; ^6^Department of Otorhinolaryngology, Head and Neck Surgery, Shanghai Ninth People’s Hospital, Shanghai Jiao Tong University School of Medicine, Shanghai, China

**Keywords:** context use, reliance on context, aging, working memory, speech understanding

## Abstract

**Purpose:**

To elucidate how aging would affect the extent of semantic context use and the reliance on semantic context measured with the Repeat–Recall Test (RRT).

**Methods:**

A younger adult group (YA) aged between 18 and 25 and an older adult group (OA) aged between 50 and 65 were recruited. Participants from both the groups performed RRT: sentence repeat and delayed recall tasks, and subjective listening effort and noise tolerable time, under two noise types and seven signal-to-noise ratios (SNR). Performance–Intensity curves were fitted. The performance in SRT50 and SRT75 was predicted.

**Results:**

For the repeat task, the OA group used more semantic context and relied more on semantic context than the YA group. For the recall task, OA used less semantic context but relied more on context than the YA group. Age did not affect the subjective listening effort but significantly affected noise tolerable time. Participants in both age groups could use more context in SRT75 than SRT50 on four tasks of RRT. Under the same SRT, however, the YA group could use more context in repeat and recall tasks than the OA group.

**Conclusion:**

Age affected the use and reliance of semantic context. Even though the OA group used more context in speech recognition, they failed in speech information maintenance (recall) even with the help of semantic context. The OA group relied more on context while performing repeat and recall tasks. The amount of context used was also influenced by SRT.

## Introduction

Speech recognition requires listeners to access phonological information and match it to its representation from semantic long-term memory (Ronnberg et al., 2019) and integrates these representations into meaningful and comprehensible sentences (Schurman et al., 2014). Both auditory and cognition systems play important roles during communication (Arlinger et al., 2009). However, aging could affect both auditory and cognitive processing in elderly listeners, manifested with effortful listening and even communication avoidance, especially under noisy conditions. One cognitive skill that could support communication in such environments is the ability to use context (Sheldon et al., 2008; Benichov et al., 2012).

Context is a general term of cues, including linguistic information like semantics, lexicon, syntactic structure, speech rate, and emotional information. It facilitates speech understanding by narrowing the lexicon search space (Janse and Jesse, 2014), guessing the missing information (Kathleen Pichora-Fuller, 2008), and accelerating word retrieval (Kave and Goral, 2017), to partially compensate for the noise interference (Janse and Jesse, 2014) and lexicon ambiguity (Kathleen Pichora-Fuller, 2008). The ability to use context is affected by the individual’s auditory-cognitive ability, including short-term/working memory (Zekveld et al., 2013; Gordon-Salant and Cole, 2016), semantic long-term memory (Ronnberg et al., 2019), vocabulary knowledge from crystal intelligence (Salthouse, 2012), and the external sound environment(Winn and Moore, 2018; Signoret and Rudner, 2019), such as the type of background noise and signal-to-noise ratios (SNR).

Age affects the use of context; however, the extent of this effect is not clear. It has been shown that crystal intelligence, which provides vocabulary and linguistic knowledge to construct and utilize context, is preserved and even improved with age (Salthouse, 2010). One can say that older adults are quite adept at and skilled in using context because they rely on it to solve their daily communication difficulties (Dubno et al., 2000; Grady, 2000). On the other hand, the process of using context taps into cognitive functions like working memory (WM) (Janse and Jesse, 2014) that may decline with age. WM helps listeners use context to constrain the semantics of speech representation and accelerate semantic integration (Zekveld et al., 2011) to compensate for the increased processing needs when the signal is degraded (Zekveld et al., 2012; Janse and Jesse, 2014; Ronnberg et al., 2019). Thus, mixed results were seen when examining the effect of age (Ronnberg et al., 2019). For example, some studies concluded that the elderly used more context than, or at least as much as, their younger counterparts during speech recognition tests in quiet and in noise (Wingfield et al., 1994; Dubno et al., 2000; Sheldon et al., 2008). Other studies, such as Jiang et al. (Jiang et al., 2017), found that younger adults may use more context than older adults during sentence recognition in noise.

The mixed results of aging on context use may originate from the different noise conditions used in the various studies as well. As stated earlier, construction and utilization of context rely on the quality of speech signal (Pichora-Fuller et al., 1995). It has been shown that context would assist speech recognition only when the SNR becomes low but not too low (Schiller et al., 2020). When the SNR is extremely favorable, context is not needed for recognition because of the high quality of the speech signal (Pichora-Fuller et al., 1995; Nagaraj, 2017). As to noise types, Van Engen et al. (Van Engen et al., 2014) and Nittrouer et al. (Nittrouer and Boothroyd, 1990) found that babble noise is more difficult than steady-state noise because of informational masking. To minimize the impact of SNRs and noise types, Kuk et al. (Kuk et al., 2020, 2021) developed a Repeat–Recall Test (RRT) as the metric to examine context use over a range of SNRs and several noise conditions.

The RRT is a more comprehensive way to assess semantic context use. It is a sentence test that includes a Repeat and a Recall task. The Repeat task is an immediate recall task that asks participants to repeat the sentence immediately after hearing it. Performance on the Repeat task mainly relies on surface phonological morphosyntax information and short-term memory (Rummer and Engelkamp, 2003; Campoy and Baddeley, 2008; Tan and Ward, 2008; Polisenska et al., 2014). The Recall task is a time-limited (1 min) delayed (15 s after sentences presentation) free-recall task. It taps into more semantic interpretation and language processing (Polisenska et al., 2014), such as rehearsal and grouping strategy (Cowan, 2001; Bunting et al., 2006). Both Repeat and Recall tasks can be used to assess semantic context use (Kuk et al., 2020). High- and low-context sentences are created using the same words and syntactical structure. A high-context (HC) sentence is semantically and syntactically correct. A low-context (LC) sentence is created from the HC sentences within the same list by moving the keywords randomly across sentences so that the sentences are no longer meaningful but are still syntactically the same. Subjective listening effort (LE) and noise tolerable time (TT) are also assessed in the RRT. The difference in performance between high- and low-context sentences represents semantic context use (CU).

In the current study, we used the Chinese-RRT to examine how age would affect semantic CU and how this could be further influenced by test conditions. We hypothesized the following: (1) Age would influence CU in four RRT tasks and the reliance on context. (2) Noise conditions would also influence CU and the reliance on context. (3) CU and the reliance on the context under different speech recognition rates (or SNRs) might be different.

## Materials and methods

### Participants

Two groups of normal-hearing adults were recruited online *via* the Department’s website. Fifty-four participants between 18 and 25 years of age (21.47 ± 2.20) were recruited into the young adult (YA) group. Fifty-two participants between 50 and 65 years of age (55.79 ± 5.23) were recruited into the older adult (OA) group. All the participants were native Mandarin speakers with audiometric thresholds < 25 dB HL from 250 to 8,000 Hz (pure tone average of YA: 10.03 ± 4.21 dB HL, OA: 20.84 ± 4.47 dB HL) and normal tympanograms. Their speech reception thresholds were tested with the Chinese HINT test, and the averages were –5.04 ± 0.88 dB for YA and –4.20 ± 0.79 dB for OA. All participants scored higher than 26 on the Montreal Cognitive Assessment (MoCA) and had over 5 years of formal education. No previous neurological diseases or long-term untreated chronic diseases were reported.

This study was approved by the Ethics Committee of the Peking University First Hospital (#2020-095). All participants signed informed consent and were financially reimbursed for their participation.

### Description of the Chinese Repeat–Recall Test

The Chinese version of the RRT was created following the same procedures as the English version (Slugocki et al., 2018; Kuk et al., 2021). It has two themes: Food and Cooking and Daily Lives, targeting a third- to fourth-grade reading level. In each theme, seven lists, each with six sentences, were available. The high-context sentences were constructed with 9–12 Chinese words per sentence, each containing 3–4 keywords. All the keywords belong to Popularized Graded Words in the classification of syllabic Chinese words for International Chinese Education. A total of 20 keywords were scored in each list of six sentences. The low-context sentences were created by reassigning the keywords of high-context sentences to other sentences within the same list while maintaining the syntactic structure of the sentences. Examples of two of the high-context sentences in a list of six sentences: 香甜的点心是用蜂蜜做的(**Sweet snacks** are made of **honey**)and 树上的橘子 成熟了(The **orange** on the **tree** is **ripe**). Examples of two of the low-context sentences in a list of six sentences:成熟的树是用橘子做的(**Ripe tree** are made of **orange**) and 点心上的蜂蜜 香甜了(The **snack** on the **honey** is **sweet**). The whole example list is shown in the Supplementary Material. The sentences were recorded by a native Chinese female professional announcer in a standard soundproof room. The speech materials were equalized to have the same root-mean-square (rms) amplitude. Speech materials were presented at a fixed 75 dB SPL in all conditions.

Two types of background noise [Two-Talker Babble (TTB) and Speech-Shaped Noise (SSN)] were also available. TTB was created by mixing the speech (from an audiobook) read aloud by two female announcers and equalizing their maximum rms level and was presented from the front (0 degrees). The SSN was created by filtering a broadband noise with a filter that has the same long-term spectrum as the speech materials and was presented from the back (180 degrees). The noise level was varied to result in SNRs of –10, –5, 0, 5, 10, 15 dB, and quiet. All the stimuli could be acquired from the online Supplementary Material.

### Repeat–Recall Test procedure

The test was performed in a sound-attenuated booth (ambient noise level <30 dB A). Speech and noise stimuli were presented *via* loudspeakers (Yamaha HS5) placed 1 m in front and behind the participant, at ear level. Instructions on the test were provided and the participants were trained with a non-test list at SNR = 10 dB before data collection. The order of RRT themes (2) and noise types (2) were counterbalanced across participants. Each of the seven sentence lists was tested at a different SNR in random order.

The test flow was the same as the English version (details in Supplementary Figure 1). Participants repeated each sentence after it was presented. Only keywords were scored when repeated correctly. The time interval between sentences was fixed at 2 s. After all six sentences were repeated, participants paused for 15 s and were instructed to recall as many of the sentences (or fragments of them) as they could within a minute. Only keywords that were repeated correctly during the Repeat phase were credited during Recall Keywords recalled correctly were scored. Afterward, participants were asked to rate how effortful it was for them to hear the sentences in the specific noise condition, which was also known as Listening Effort (LE). To evaluate LE, a visual analog scale (VAS) from 1 to 10 was used, with “1” as the least effortful and “10” as the most effortful. A rating of “11” was allowed if participants gave up because of the extremely noisy condition. Afterward, participants estimated the amount of time (in minutes) they were willing to spend communicating under the specific test condition, which is also known as Tolerable Time (TT). The low-context sentences were always presented before the high-context sentences to minimize any learning effect. A total of 28 trials (2 noises * 7 lists (SNRs) * 2 context conditions) were completed in one 1-h session. Rests were provided whenever needed to make sure both age groups, especially OA, would keep their attention and not be fatigued.

### Data processing and statistical analysis

In the current study, CU was calculated as the difference between high- and low-context sentences. Since Repeat and Recall were scored as percent-correct, CU of Repeat and Recall were also transformed into rationalized arcsine unit (RAU) for further analysis (Studebaker, 1985). As CU was a difference-based index, a higher-context performance and a lower-context performance may lead to a similar CU, but with a different interpretation or implication. A high-context sentence represents a regenerated lexicon combination with helpful semantic (Potter and Lombardi, 1990). A low-context sentence simulates a word string relying on phonological and semantic representations (Haarmann et al., 2003). We also introduced another index: Proportion of CU (PCU) for Repeat and Recall separately. PCU was calculated as the proportion of CU to high-context scores (both CU and high-context scores were raw data without transforming to RAU), to measure how much one relied on context when repeating and recalling high-context sentences.

The Performance-Intensity (PI) curves of Repeat, Recall, LE, and TT were first simply drawn with the primary data. Then, smooth PI curves were fitted in the same way as done by Yang et al. (Zhigang Yang, 2007). The smooth curves of Repeat and Recall drawn through these points were logistic functions of the form


$$y=\frac{100}{1+e^{-\sigma \,\left(x-\mu \right)}}$$

The smooth curves of LE drawn through these points were logistic functions of the form


$$y=\frac{10}{1+e^{-\sigma \,\left(x-\mu \right)}}$$

The smooth curves of TT drawn through these points were logistic functions of the form


$$y=\frac{120}{1+e^{-\sigma \,\left(x-\mu \right)}}$$

For all these curves, *y* was the probability of correctly repeating or recalling the keyword, *x* was the SNR corresponding to *y*, μ was the SNR corresponding to 50% correct on the psychometric function, and σ determined the slope of the psychometric function. The parameters (μ and σ), which were used to generate the curves in Supplementary Figures 2, 3, minimized Pearson’s χ^2^ goodness of fit of the model to the data. Quiet was treated as SNR = 30 dB when fitting the curves. SNRs corresponding to 50% (SRT50) and 75% (SRT75) speech recognition rate of high-context sentences in the Repeat task was calculated. Then, the corresponding performances for Repeat in low-context, Recall, LE, and TT in both high- and low-context were identified under the two SNRs. P/CU(Repeat_50%/ 75%_), P/CU(Recall_50%/75%_), CU(LE_50%/75%_), and CU(TT_50%/75%_) were calculated in the same way as aforementioned using the raw data.

The SPSS 25.0 software was used for the data analysis. A mixed-design was adopted with age groups as between-subject factors, while noise types, SNRs, and context as within-subject factors. Due to the skewed distribution of the data and repeated measurement, Generalized Linear Mixed Model (GLMM) was first applied to analyze the fixed (and interaction) effects of context (high vs. low) and age (younger vs older) to target variables: Repeat (in RAU), Recall (in RAU), and TT with random effects included an intercept for each participant. LE was a rank variable so that Generalized Estimated Equation (GEE) was performed to analyze the fixed effects. The effects of Age groups and Noise types on the target variables (CU (Repeat, Recall, and TT) and PCU (Repeat and Recall)) were also analyzed with GLMM, with random effects included as intercepts for each participant. Target variable CU(LE) was analyzed with GEE similarly. Factors with significant fixed effects were further analyzed for interaction effects. All the GLMM and GEE analyses were corrected for multiple comparisons using sequential Bonferroni. Degrees of freedom was fixed for all tests with a residual method. Independent sample *t*-tests were performed to further compare differences between groups at separate SNRs. Wilcoxon tests were performed to check whether P/CU under SRT50 was significantly different from that under SRT75 in the same age group. Mann Whitney *U* tests were performed to check whether P/CU in OA was significantly different from that in YA under the same SRT condition. Outliers were identified using a Box plot in SPSS and were excluded when performing the tests. A two-tailed *p* < 0.05 was considered statistically significant.

## Results

### Performance-Intensity functions of Repeat–Recall Test and P/CU

**FIGURE 1:**
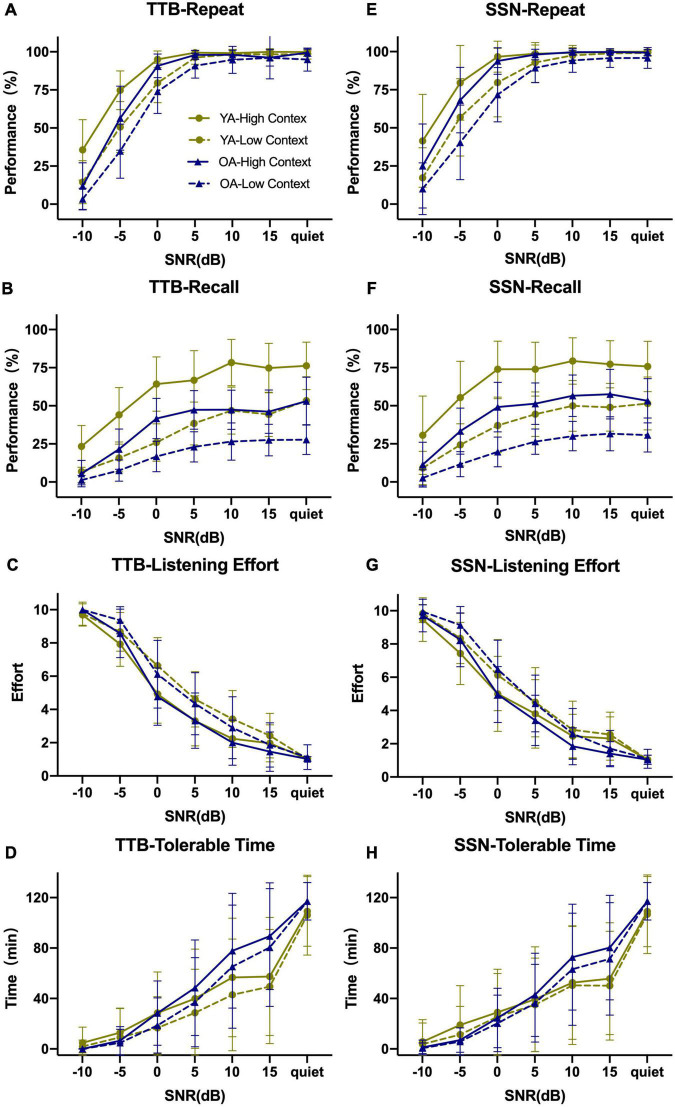
Overview of Repeat–Recall Test (RRT) performance in two-talker babble (TTB) and speech-shaped noise (SSN) for both age groups in seven SNRs. **(A–D)** Performance for TTB; **(E–H)** performance for SSN. Both performances for Repeat and Recall would increase as SNR became high, along with decreasing LE and prolonging TT. Recall seemed more insensitive to SNR comparing with Repeat and the curves were more smoothy than Repeat. Error bars represented one standard deviation.

**FIGURE 2:**
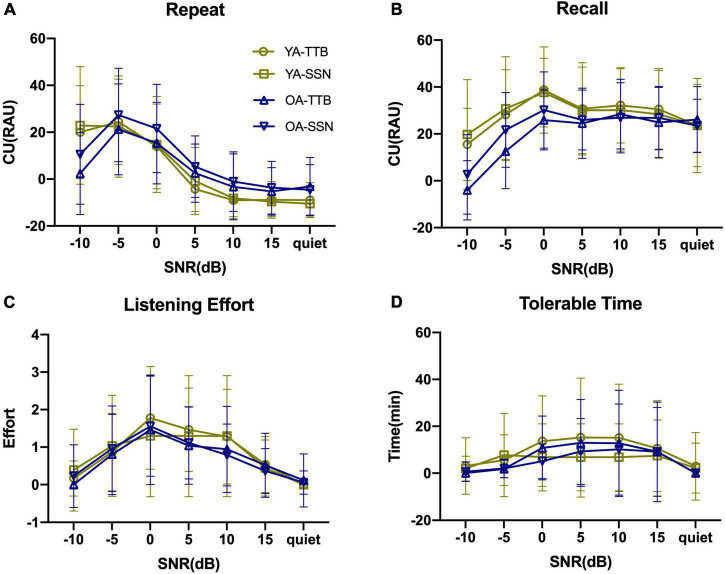
Context use (CU) of two age groups under two noise conditions and seven SNRs. **(A)** As for CU (Repeat), both age groups increased as SNR becomes worse and OA finally decreased when SNR = –10 dB, where YA could still hold the utilization of context. **(B)** As for CU (Recall), both age groups maintained their utilization of context when SNR > –5 dB and started to decrease at SNR = –5 and –10 dB. **(C)** As for CU (LE), LE was alleviated by context, and such alleviation arose as SNR became worse, peaking at SNR = 0 dB, and then decreased. **(D)** As for CU(TT), it seemed that it had a more stable trend as SNR changed. Error bars represented one standard deviation.

**FIGURE 3:**
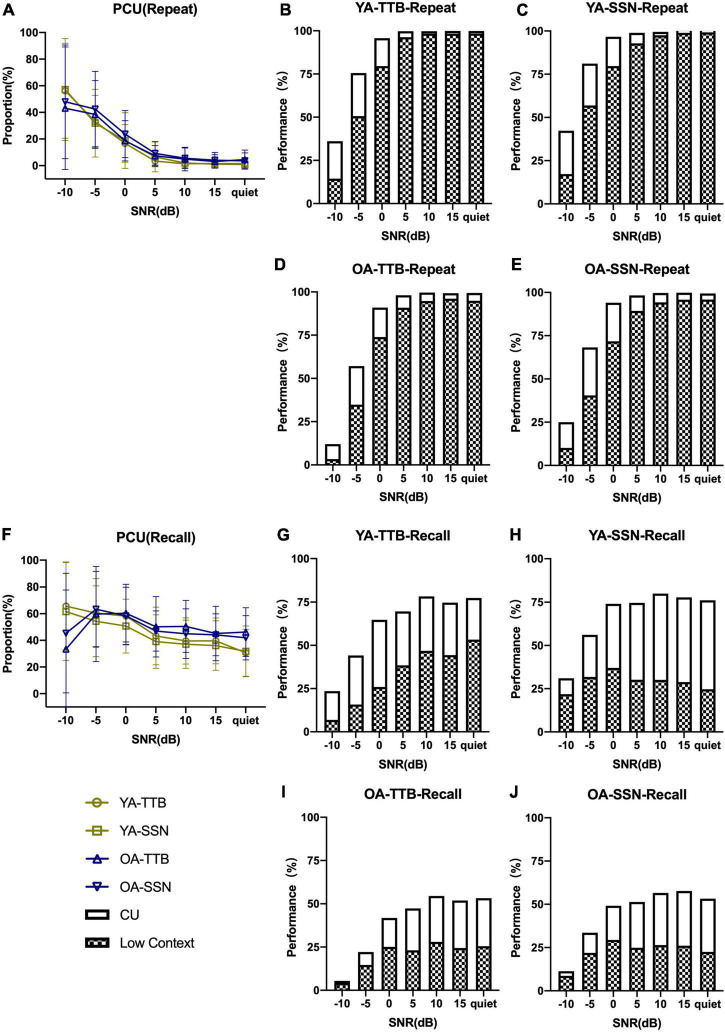
Proportion of CU in Repeat and Recall of two age groups under two noise conditions and seven SNRs. **(A)** PCU (Repeat) gradually rose as SNR became even worse. **(B–E)** PCU (Repeat) in two age groups under two noise types. **(F)** PCU (Recall) also rose as SNR decrease but in a much smooth trend, and OA failed to rely on context any more in SNR = –10 dB, where acoustic signals were degraded seriously. **(G–J)** PCU(Recall) in two age groups under two noise types. Hollow bars represented CU and grid represented low-context performance. The combination of the two represented the performance of high-context. Error bars represented one standard deviation.

Figure 1 displays the PI functions of RRT in the two age groups (two contexts and two noise conditions) with raw data. P/CU were calculated and PI functions were displayed in Figures 2, 3. The fitted PI curves are shown in the Supplementary Figures 2, 3.

Context significantly affected all the four tasks in RRT (Repeat: *F*_(1,2966)_ = 376.924, *P* < 0.001; Recall: *F*_(1,2966)_ = 1268.480, *P* < 0.001; LE: Wald χ^2^(1) = 187.394, *P* < 0.001; TT: *F*_(1,2966)_ = 169.258, *P* = 0.004). It improved Repeat and Recall performance, lowered subjective listening effort, and increased tolerable time. Age also significantly affected Repeat, Recall, and TT (Repeat: *F*_(1,2966)_ = 46.407, *P* < 0.001; Recall: *F*_(1,2966)_ = 706.315, *P* < 0.001; TT: *F*_(1,2966)_ = 8.380, *P* = 0.004). The interaction between context and age was found to be significant (Repeat: *F*_(3,2964)_ = 163.208, *P* < 0.001; Recall: *F*_(3,2964)_ = 872.296, *P* < 0.001; TT: *F*_(3,2964)_ = 59.543, *P* < 0.001). These results confirmed that participants in different age groups performed differently in different contexts (Table 1).

**TABLE 1:** Fixed effects of context, age group, and interaction effects to RRT tasks.

		Context	Age	Context*Age^a^
Repeat	F	376.924***	46.407***	163.208***
	df1	1	1	3
	df2	2966	2966	2964
Recall	F	1268.480***	706.315***	872.296***
	df1	1	1	3
	df2	2966	2966	2964
LE	Wald χ^2^	187.394***	0.560	–
	df	1	1	–
TT	F	169.258***	8.380**	59.543***
	df1	1	1	3
	df2	2966	2966	2964

^a^Only factors with significant fixed effects will be further analyzed with interaction effects. **P < 0.01, ***P < 0.001.

### Age effect on P/CU

Age significantly affected CU on the Repeat, Recall, and TT tasks but not on the LE (Table 2). Results indicated that the YA group had a smaller CU (Repeat) (*F*_(1,1482)_ = 49.291, *P* < 0.001), a larger CU (Recall) (*F*_(1,1482)_ = 69.083, *P* < 0.001), and a longer CU (TT) (*F*_(1,1482)_ = 14.001, *P* < 0.001) than the OA group. In both high- and low-context conditions, LE was not significantly different between the two age groups (low-context: β = 0.038, Wald χ^2^ (1) = 0.210, *P* = 0.647; high-context: β = 0.086, Wald χ^2^ (1) = 0.921, *P* = 0.337). This also indicated that there was no significant fatigue in older adults during this 1-h session.

**TABLE 2:** Fixed effects and interaction effects to CU and PCU.

		Age	Noise type	Age*Noise type^a^
CU(Repeat)	F	49.291***	0.033	–
	df1	1	1	–
	df2	1482	1482	
CU(Recall)	F	69.083***	1.072	–
	df1	1	1	–
	df2	1482	1482	
CU(LE)	Wald χ^2^	0.184	3.386	–
	df	1	1	–
CU(TT)	F	14.001***	8.076***	6.671**
	df1	1	1	3
	df2	1482	1482	1480
PCU(Repeat)	F	58.623***	0.404	–
	df1	1	1	–
	df2	1482	1482	
PCU(Recall)	F	38.783***	5.755*	14.637***
	df1	1	1	3
	df2	1482	1482	1480

^a^Only factors with significant fixed effects will be further analyzed with interaction effects. *P < 0.05, **P < 0.01, ***P < 0.001.

Results showed that the OA group had a higher PCU than the YA group on both Repeat (*F*_(1,1482)_ = 58.623, *P* < 0.001) and Recall (*F*_(1,1482)_ = 38.783, *P* < 0.001) tasks. This suggested higher reliance on the semantic context in OA than YA group (Table 2).

### Noise types effect on P/CU

Noise types significantly affected CU (TT) with a longer time in TTB than SSN (*F* (_1,1482)_ = 8.076, *P* = 0.005). PCU (Recall) was larger in TTB than SSN (*F*_(1,1482)_ = 5.755, *P* = 0.017) (Table 2). However, noise types failed to affect P/CU (Repeat), CU (Recall), or CU (LE) (*P* > 0.05).

The interaction effect of age and noise type on CU (TT) was also significant (*F*_(3,1480)_ = 6.671,*P* = 0.001). CU (TT) was similar for the two noise types in OA (Coefficient = 0.768, *t* = 0.942, *P* = 0.346). However, it was significantly longer for TTB than for SSN in YA (Coefficient = 2.429, *t* = 3.037, *P* = 0.002). The interaction effect of age and noise type on PCU (Recall) was also significant (*F*_(3,1480)_ = 14.637,*P* < 0.001). PCU (Recall) was similar for the two noise types in OA (Coefficient = 0.021, *t* = 1.303, *P* = 0.193); however, it was significantly larger for TTB than for SSN in YA (Coefficient = 0.032, *t* = 2.000, *P* = 0.046). Details are shown in Supplementary Table 1.

### P/CU at different speech recognition rates and signal-to-noise ratios

The SRT50 and SRT75 were identified by fitted PI curves: SRT50 was 5.71 ± 2.10 dB for OA-TTB, 7.50 ± 3.78 dB for OA-SSN, 8.59 ± 2.33 dB for YA-TTB, 8.47 ± 3.58 dB for YA-SSN. SRT75 was 3.52 ± 1.93 dB for OA-TTB, 5.21 ± 3.01 dB for OA-SSN, 5.44 ± 1.73 dB for YA-TTB, 6.35 ± 3.78 dB for YA-SSN.

**FIGURE 4:**
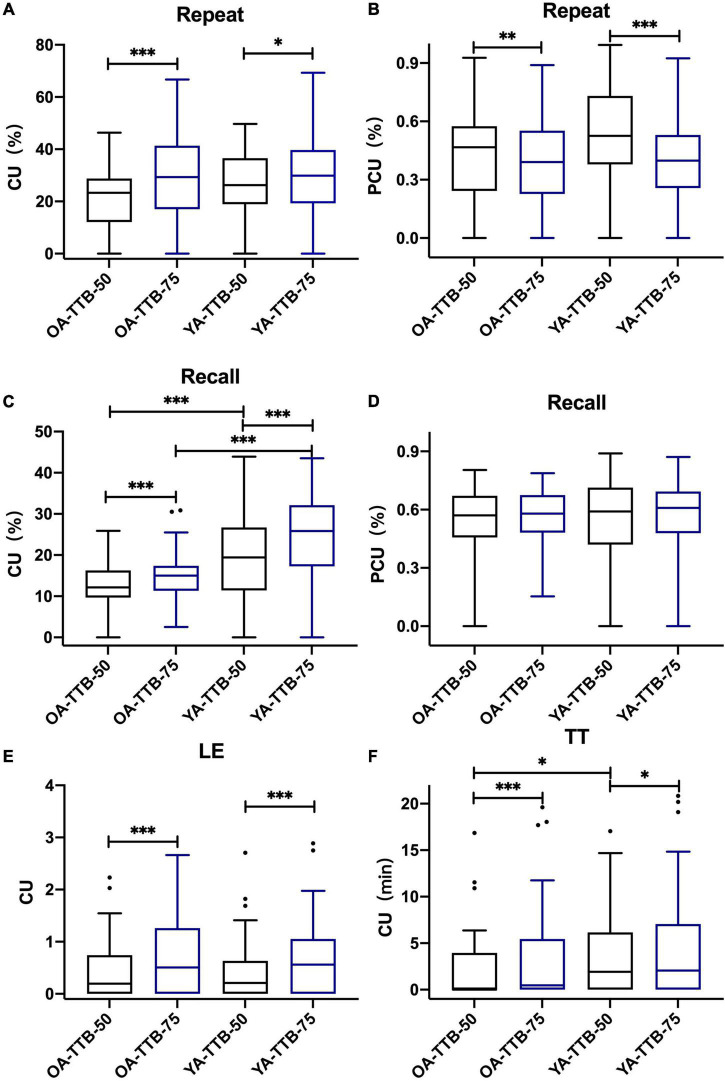
P/CU performances under two SRTs in two age groups in TTB. **(A)** CU (Repeat_50%/75%_), **(B)** PCU (Repeat_50%/75%_), **(C)** CU(Recall_50%/75%_), **(D)** PCU (Recall_50%/75%_), **(E)** CU (LE_50%/75%_), **(F)** CU (TT_50%/75%_). All the figures were Boxplot. The top and bottom lines of a column represented the maximum and minimum values of the data, respectively. The top and bottom lines of the box represented the third quartile and the first quartile, respectively, and the line in the middle of the box represents the median of the data. Black circles represented outliers. *P < 0.05, ^**^P < 0.01, ^***^P < 0.001.

**FIGURE 5:**
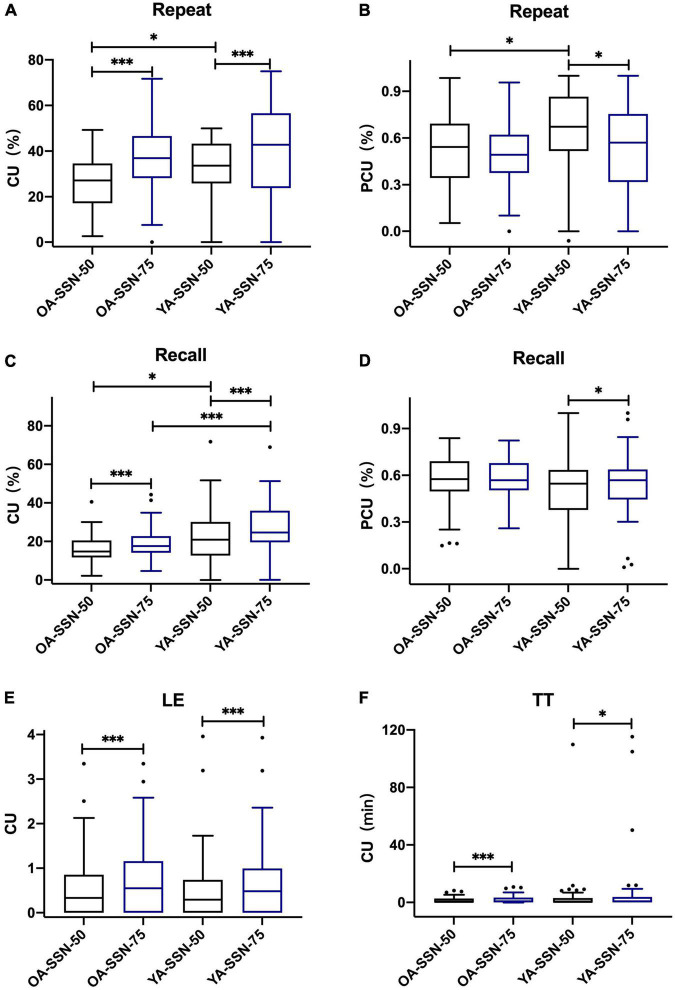
P/CU performances under two SRTs in two age groups in SSN. **(A)** CU (Repeat_50%/75%_), **(B)** PCU (Repeat_50%/75%_), **(C)** CU (Recall_50%/75%_), **(D)** PCU (Recall_50%/75%_), **(E)** CU (LE_50%/75%_), **(F)** CU (TT_50%/75%_). All the figures were Boxplot. The top and bottom lines of a column represented the maximum and minimum values of the data, respectively. The top and bottom lines of the box represented the third quartile and the first quartile, respectively, and the line in the middle of the box represents the median of the data. Black circles represented outliers. *P < 0.05, ^***^P < 0.001.

For both age groups, the results indicated that in TTB, CUs in SRT50 were significantly lower than their counterparts in SRT75 with *P* < 0.05 (Figure 4 and Table 3). PCU (Repeat_50%_) was significantly higher than PCU (Repeat_75%_) for both age groups. PCU (Recall_75%_) was similar to PCU (Recall_50%_) in both age groups. In SSN, CUs in SRT50 were significantly lower than their counterparts in SRT75 with *P* < 0.05 (Figure 5 and Table 3). PCU of OA was the same in both SRTs. However, in YA, PCU (Repeat_50%_) was significantly lower than PCU (Repeat_75%_).

**TABLE 3:** Differences between SRT75 and SRT50 in two age groups under two noise types.

		TTB	SSN
		Z	Z
OA	CU(Repeat_75%_)-CU(Repeat_50%_)	−4.406***	−5.883***
	PCU(Repeat_75%_)-PCU(Repeat_50%_)	−2.681**	−1.530
	CU(Recall_75%_)-CU(Recall_50%_)	−6.215***	−6.193***
	PCU(Recall_75%_)-PCU(Recall_50%_)	−1.153	−0.301
	CU(LE_75%_)-CU(LE_50%_)	−4.119***	−5.012***
	CU(TT_75%_)-CU(TT_50%_)	−4.088***	−4.553***
YA	CU(Repeat_75%_)-CU(Repeat_50%_)	−2.333*	−4.016***
	PCU(Repeat_75%_)-PCU(Repeat_50%_)	−4.980***	−2.293
	CU(Recall_75%_)-CU(Recall_50%_)	−6.220***	−5.782***
	PCU(Recall_75%_)-PCU(Recall_50%_)	−1.084	−2.398*
	CU(LE_75%_)-CU(LE_50%_)	−5.359***	−4.402***
	CU(TT_75%_)-CU(TT_50%_)	−2.430*	−2.812**

*P < 0.05, **P < 0.01, ***P < 0.001.

The results also showed that under both SRT50 and SRT75, CU (Recall_50%/75%_) was significantly higher in YA than OA in both noise types (*P* < 0.05). Moreover, in SSN, P/CU(Repeat_75%_) was higher in YA than OA (*P* < 0.05). Details are shown in Table 4.

**TABLE 4:** Differences between two age groups in SRT50 and SRT75.

		SRT50	SRT75
		Z	Z
TTB	CU(Repeat)	−1.760	−0.095
	PCU(Repeat)	−1.760	−0.095
	CU(Recall)	−3.697***	−4.967***
	PCU(Recall)	−0.265	−0.638
	CU(LE)	−0.085	−0.330
	CU(TT)	−2.331*	−1.361
SSN	CU(Repeat)	−2.305*	−0.882
	PCU(Repeat)	−2.305*	−0.882
	CU(Recall)	−2.266*	−4.076***
	PCU(Recall)	−1.567	−1.024
	CU(LE)	−0.367	−0.480
	CU(TT)	−0.981	−0.673

*P < 0.05, ***P < 0.001.

Independent *t*-tests were performed to evaluate the difference in P/CU (Repeat, Recall) between two age groups across SNRs while collapsing performances from both noise conditions (Supplementary Table 2). The results showed that (1) CU (Repeat) for OA was significantly higher than that for YA at SNR ≥ 5 dB (*P* < 0.001). Differences between age groups disappeared at SNR = 0, 5 dB (*P* > 0.05). When SNR = –10 dB, CU (Repeat) for OA was significantly lower than that for YA (*t* (104) = 4.974, *P* < 0.001). (2) CU (Recall) for the two age groups was similar when SNR ≥ 10 dB (*P* > 0.05), but higher for YA than OA at SNR ≤ 5 dB (*P* < 0.05). (3) Both PCU (Repeat) and PCU (Recall) for OA were significantly higher than those for YA when SNR ≥ 5 dB (*P* ≤ 0.01). PCU(Recall) was significantly lower in OA when SNR = 10 dB (*t* (89.573) = 4.13, *P* < 0.001).

## Discussion

In the current study, we examined semantic context use (CU) and reliance on semantic context (PCU) in two age groups under two noise types on the various measures of the Chinese RRT (Repeat, Recall, Listening Effort, and Tolerable Time) over a range of SNRs. As expected, CU and PCU were significantly influenced by age. OA used more semantic context in the Repeat task and relied more on the semantic context in both Repeat and Recall tasks than YA. In contrast, YA used more semantic context in the Recall and Tolerable Time tasks and relied less on semantic context than OA. Context use in SRT50 was significantly lower than that in the SRT75 for both the age groups in the four RRT tasks. The reliance on the context in the Repeat task was also higher in SRT50 than in SRT75. At the same SRT, context use in Repeat (SRT50 in SSN only) and Recall tasks were significantly higher in YA than those in OA.

### Cognitive processes of semantic context use in repeat and recall tasks

The Repeat task can be viewed as a process of context acquisition, where speech signals that provide (or do not) a semantic context are gathered (Rummer and Engelkamp, 2003; Campoy and Baddeley, 2008; Tan and Ward, 2008; Polisenska et al., 2014). As such, it is more susceptible to the impact of poor SNR than Recall when SNR ≤ 0 dB (Figures 1A,E vs. Figures 1B,F, respectively). In the current study, when SNR > 0 dB, minimal CU was observed for Repeat (Pichora-Fuller et al., 1995; Nagaraj, 2017). The Recall task is a process of semantic context maintenance (Barrett et al., 2004), which is less affected by SNR (once it is audible), but more affected by cognitive function than Repeat. Indeed, CU (Recall) was maintained at a high level above SNR = 0 dB. This was also consistent with the nature of Recall as semantic interpretation and language processing (Polisenska et al., 2014).

We believe that both Repeat and Recall tasks benefit from WM since it is a fundamental cognitive function related to speech understanding (Baddeley, 2012) and online sentence processing (Evans et al., 2015). The Ease of Language Understanding (ELU) model (Ronnberg et al., 2019) acknowledges that when a mismatch between phonological and semantic information occurs during the explicit process, WM helps to construct a supportive context. Because WM is a limited resource, it is shared between information processing and storage (Baddeley, 2012). During the Repeat task of context acquisition, more WM is allocated to processing. During the Recall task of context maintenance, more WM is allocated to storage (Tan et al., 2017). CU on the two tasks would likely be complementary (i.e., as one increases, the other decreases). Once resource demand exceeds its capacity limit, performance decreases. This may be evident from the complementary changes in CU for the Repeat and Recall tasks across SNRs from quiet to SNR = –5 dB. At SNR = –10 dB, CU was the poorest in the OA who are likely limited in WM.

### How does age affect semantic context use and context reliance?

In the current study, we found that OA performed more poorly in the low-context sentences than YA in the Repeat task leading to a higher P/CU (Repeat) (low-context coefficient = –6.828; high-context coefficient = –1.183, *P* < 0.001; Table 5 for statistic details). As repeating low-context sentences rely on phonological maintenance and rehearsal (Bailey et al., 2009), the declined processing speed (Salthouse, 2000; Arlinger et al., 2009) in OA makes it harder for them to match phonological information to representation accurately and efficiently without semantic context. Due to the relatively low WM capacity (confirmed by the Backward Digit Span task, where the performance of YA was 8.59 ± 2.25 and that of OA was 5.75 ± 1.74, *t* (104) = 7.204, *P* < 0.001), declined semantic short-term memory (Haarmann et al., 2003) in the OA may be another explanation. This explanation also aligns with the finding that when the context is unavailable, the reliance on working memory increases (Nagaraj, 2017), and participants with higher WM can better handle low-context sentences (Moradi et al., 2014) than those with lower WM. This result is consistent with a previous study by Sheldon et al. (2008), which showed that context helped reduced the number of noise-vocoded bands needed for 50% word recognition, and this reduction was more in OA than YA. Aydelott et al. (2010) showed that under quiet conditions, OA used more context than YA in word recognition in quiet. Our study further expanded the range of SNR and explained how CU (Repeat) is influenced by SNR. When SNR ≥ 5 dB, OA had significantly higher CU (Repeat) than YA; this difference disappeared as SNR became poorer (SNR = 0, 5 dB) and reversed when SNR = −10 dB. This suggested that YA could use more semantic context only when it is needed at unfavorable SNR. This observation was supported by the PCU (Repeat) PI function that showed a steep negative slope at SNR < 0 dB. This also implied that CU (Repeat) relies on the demand for semantic context, and if the test condition was unfavorable, YA could use context equally or more than OA (Dubno et al., 2000; Aydelott et al., 2010; Jiang et al., 2017).

**TABLE 5:** Interaction effects of age groups * Context in Repeat and Recall.

	OA-YA low context	OA-YA high context
	Coefficient	Coefficient
Repeat	−6.828***	−1.183***
Recall	−17.082***	−22.520***

***P < 0.001.

In the current study, we also found that OA performed poorer than YA in the Recall of high-context sentences. This led to lower CU (Recall) and higher PCU (Recall) in OA than YA (low-context: Coefficient = 17.082; high-context: Coefficient = −22.520, *P* < 0.001; Table 5 for statistic details). Recall of high-context sentences relies on semantic maintenance (Potter and Lombardi, 1990; Bailey et al., 2009) and can be considered understanding a regenerated lexicon combination with helpful semantic (Potter and Lombardi, 1990). It has been proved that WM helps semantic integration (Zekveld et al., 2011; Yang et al., 2020) by accelerating word retrieval (Unsworth et al., 2012), but was declined in our sample (compared to the YA sample). Besides, the semantic strategy also declines with aging (Haarmann et al., 2005). In addition, since OA is more easily distracted due to a lack of inhibitory control and attention (Hasher et al., 1991; Peelle et al., 2010; Salthouse, 2012), which can also benefit from WM (Baddeley, 2003; Barbas et al., 2018), the noise during the 15 s retention and the 1 min Recall process would increase the burden of maintaining information (both semantic context information from high-context sentences, and lexicon information from low-context sentences) for OA. YA with higher working memory may have more storage resources for even low-context recall. Accordingly, YA could use more semantic context than OA in Recall. Golomb et al. (2008) showed similar results that YA used more context to help visual recall of words than OA. As we expanded the SNR range, we found that the difference between age groups disappeared when SNR > 5 dB. This was because the noise was not distracting enough to interfere with context maintenance. Tun et al. (2002) also concluded that there was no difference between YA and OA in quiet conditions when recalling high-context text and low-context word strings. This means that when speech audibility is ensured, both groups could use semantic context similarly when recalling, even though OA may rely more on it. When SNR ≤ 5 dB, YA could make more use of semantic context than OA.

We failed to see the effect of age on CU (LE). This is in line with the result from Hunter et al. (Hunter and Humes, 2022), who interpreted that OA is quite an expert in context utilization, and this process seems to happen automatically without consuming extra effort. Thus, the two age groups might reduce the same amount of LE when providing context.

We found that age affected CU (TT) and that semantic context would prolong noise tolerable time more in YA. This may suggest that OA are quite familiar with the suboptimal listening environment, and tolerable time is less affected by context (Pichora-Fuller and Singh, 2006) than in YA.

### How does signal-to-noise ratio affect semantic context use and context reliance?

In the current study, CU in SRT75 was significantly higher than that in SRT50. This indicates that a relatively higher quality of speech signal was conducive to constructing a context and utilizing it in different tasks (Pichora-Fuller et al., 1995). Also, the reliance on context may also be lower, and this seemed to be more prominent in Repeat. Reliance on the context during Recall seemed less affected by SRT.

In SRT75, which is closer to the daily situation than SRT50, P/CU(Repeat_75%_) was similar in both age groups. This indicated that when restricting the amount of speech information one could get, semantic CU during speech recognition for young and old adults were nearly the same. This result was different from previous studies that concerned the same test SNR and neglected the different SRT in both age groups (Wingfield et al., 1994; Dubno et al., 2000; Sheldon et al., 2008). Therefore, the difference in CU may also arise from SRT. However, YA could use more context when recalling semantic information. This could be explained by the relatively higher working memory capacity of YA to maintain more semantic information. In SRT50, we found that the difference in CU in Recall still existed between the two age groups. Besides, P/CU(Repeat) in YA was higher than that in OA in SSN but not in TTB.

## Conclusion

In the current study, we used the Chinese RRT to examine the effect of age on semantic context use and semantic context reliance under different test conditions and test items. We concluded that even though older adults may acquire more semantic context to help with repeating information, they still face difficulties in maintaining semantic context information for later recall. For both repeat and recall tasks, older adults tended to rely more on context. SRT influenced the performance of context use and reliance on the context in the two age groups, reminding us to pay attention to SRT in future research.

## Data availability statement

The raw data supporting the conclusions of this article will be made available by the authors, without undue reservation.

## Ethics statement

The studies involving human participants were reviewed and approved by the Ethics Committee of the Peking University First Hospital. The participants provided their written informed consent to participate in this study.

## Author contributions

JS collected the data, organized the database, performed the statistical analysis, and wrote the manuscript. ZZ, HL, CW, and YL contributed to the conception and design of the study. BS participated in the data collection and technical support. All authors contributed to the article and approved the submitted version.

## Conflict of interest

BS was employed by Widex Hearing Aid (Shanghai) Co., Ltd. The remaining authors declare that the research was conducted in the absence of any commercial or financial relationships that could be construed as a potential conflict of interest.

## Publisher’s note

All claims expressed in this article are solely those of the authors and do not necessarily represent those of their affiliated organizations, or those of the publisher, the editors and the reviewers. Any product that may be evaluated in this article, or claim that may be made by its manufacturer, is not guaranteed or endorsed by the publisher.
