# Supplementary material for: The effect of aging on context use and reliance on context in speech: A behavioral experiment with Repeat–Recall Test
Source: Front Aging Neurosci. 2022 Jul 22;14:924193. doi: 10.3389/fnagi.2022.924193 (PMC9354826; doi:10.3389/fnagi.2022.924193)
Supplement: Supplementary file 1 [file Data_Sheet_1.DOCX]

***Supplementary Material***

Figure S1. Flow chart of RRT at one context*SNR*noise condition test condition. Noise started 10 s before the first sentence was presented and lasted till the end of Recall. Participants were asked to repeat after each of 6 sentences, After the last (6^th^) sentence was repeated, they paused for 15 s after which, they were asked to recall keywords as more as possible in 1 min. Subject LE and TT were evaluated at the end of a trial.


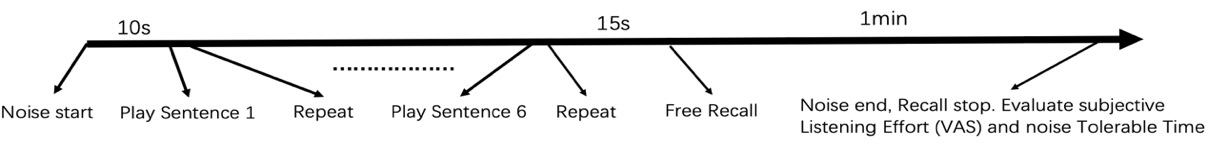

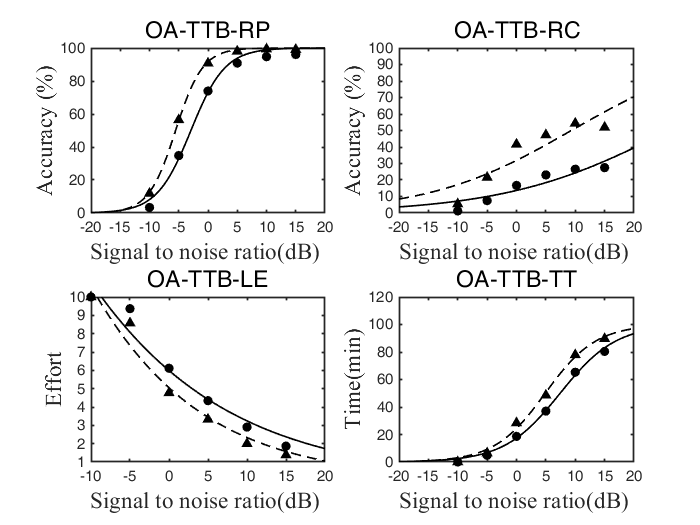

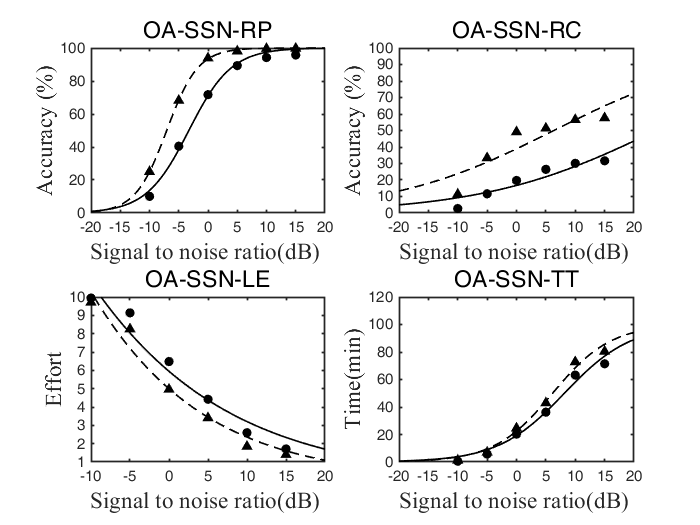


Figure S2 Fitted PI curves of OA in two noise types. A-D represented the curves of Repeat, Recall, LE, and TT in TTB. E-H represented the curves of Repeat, Recall, LE, and TT in SSN.

H

G

F

E

D

B

A

C


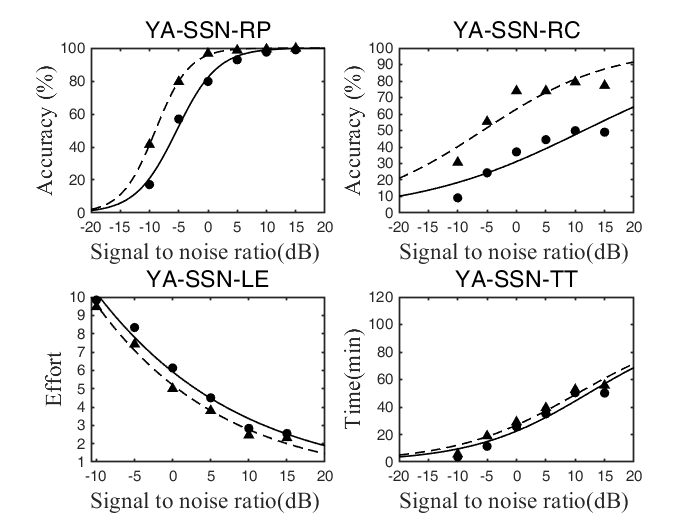

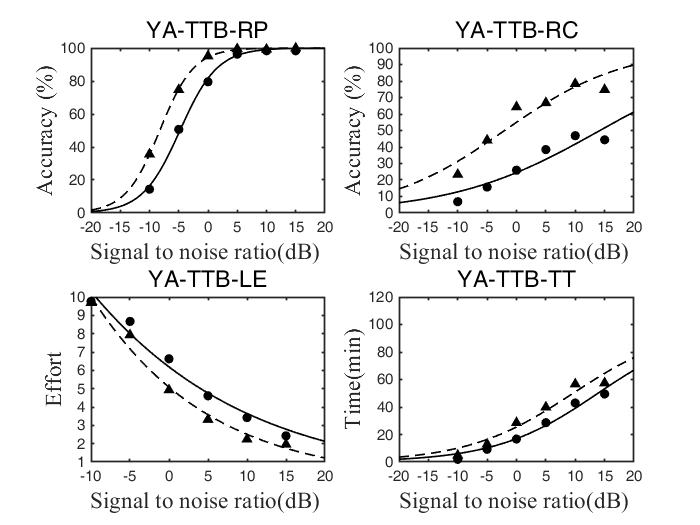


Figure S3 Fitted PI curves of YA in two noise types. A-D represented the curves of Repeat, Recall, LE, and TT in TTB. E-H represented the curves of Repeat, Recall, LE, and TT in SSN.

H

F

B

D

G

E

A

C

One example of Sentence lists of RRT in Chinese (keywords are underlined)

Theme 1: Food & Cooking

List 1-High context

香甜的点心是用蜂蜜做的

姐姐的背包里有诱人的零食

板凳上的桃子真红润

小麦是植物的种子

抽屉里的奶油 雪糕过期了

弟弟喜欢喝 冰镇的可乐

List 1-Low context

诱人的抽屉是用可乐做的

奶油的板凳里有红润的喝

点心上的植物真冰镇

姐姐是桃子的蜂蜜

弟弟里的香甜 种子过期了

小麦喜欢背包 雪糕的零食

Recordings of background noise are available in the on-line supplementary materials, and are listed here:

1. Babble Noise SNR=0 dB
2. Babble Noise SNR=5 dB
3. Babble Noise SNR=10 dB
4. Babble Noise SNR=15 dB
5. Babble Noise SNR=-5 dB
6. Babble Noise SNR=-10 dB
7. Speech Shaped Nosie SNR=0 dB
8. Speech Shaped Nosie SNR=5 dB
9. Speech Shaped Nosie SNR=10 dB
10. Speech Shaped Nosie SNR=15 dB
11. Speech Shaped Nosie SNR=-5 dB
12. Speech Shaped Nosie SNR=-10 dB
