# Supplementary material for: The effect of aging on context use and reliance on context in speech: A behavioral experiment with Repeat–Recall Test
Source: Front Aging Neurosci. 2022 Jul 22;14:924193. doi: 10.3389/fnagi.2022.924193 (PMC9354826; doi:10.3389/fnagi.2022.924193)
Supplement: Supplementary file 2 [file Data_Sheet_2.docx]

Table S1 The interaction effect of age* noise type on CU(TT) and PCU(Recall)

|  | OA-TTB vs OA-SSN | YA-TTB vs YA-SSN |
| --- | --- | --- |
|  | Coefficient | Coefficient |
| CU(TT) | 0.768 | 2.429^**^ |
| PCU(Recall) | 0.021^*^ | -0.032^*^ |

^*^: P < 0.05, ^**^: P < 0.01, ^***^: P < 0.001

Table S2. Independent T test of CU (Repeat, Recall) and PCU (Repeat, Recall) between two age groups in different SNRs

|  |  |  | YA | | OA | |  |  |
| --- | --- | --- | --- | --- | --- | --- | --- | --- |
|  |  | SNR | Mean | SD | Mean | SD | t | df |
| CU | RP | QUITE | -9.7286 | 4.96528 | -3.8801 | 8.55217 | -4.285*** | 81.245 |
|  |  | 15 | -9.2515 | 5.18613 | -4.449 | 8.09584 | -3.622*** | 86.302 |
|  |  | 10 | -8.5963 | 4.96019 | -2.1765 | 11.15517 | -3.804*** | 69.833 |
|  |  | 5 | -2.5417 | 7.98042 | 3.9049 | 10.29998 | -3.61*** | 104 |
|  |  | 0 | 14.3622 | 13.87517 | 18.4746 | 12.74222 | -1.588 | 104 |
|  |  | -5 | 23.4451 | 15.14927 | 24.3082 | 14.44197 | -0.3 | 104 |
|  |  | -10 | 21.4661 | 15.61746 | 6.4706 | 15.40918 | 4.974*** | 104 |
|  | RC | QUITE | 23.5613 | 13.72473 | 24.7968 | 9.22663 | -0.546 | 93.109 |
|  |  | 15 | 29.4461 | 12.79743 | 25.8695 | 11.61756 | 1.505 | 104 |
|  |  | 10 | 31.1628 | 11.82917 | 27.6608 | 10.24389 | 1.627 | 104 |
|  |  | 5 | 30.3819 | 13.11674 | 25.2282 | 10.12117 | 2.259* | 104 |
|  |  | 0 | 38.1473 | 11.63824 | 28.03 | 11.13953 | 4.569*** | 104 |
|  |  | -5 | 29.485 | 14.60587 | 17.1227 | 10.98778 | 4.936*** | 104 |
|  |  | -10 | 17.6532 | 14.44248 | -0.6856 | 10.72134 | 7.401*** | 104 |
| PCU | RP | QUITE | 1.16% | 1.99% | 4.10% | 5.25% | -3.783*** | 64.842 |
|  |  | 15 | 1.35% | 2.12% | 3.63% | 4.32% | -3.423** | 73.675 |
|  |  | 10 | 1.67% | 2.23% | 5.21% | 7.24% | -3.377** | 60.245 |
|  |  | 5 | 4.99% | 5.74% | 8.27% | 6.86% | -2.675** | 104 |
|  |  | 0 | 17.77% | 11.97% | 21.17% | 11.76% | -1.473 | 104 |
|  |  | -5 | 32.47% | 17.78% | 40.47% | 20.26% | -2.162* | 104 |
|  |  | -10 | 56.70% | 26.16% | 45.52 | 33.64% | 1.915 | 104 |
|  | RC | QUITE | 31.20% | 13.58% | 44.08% | 12.67% | -5.043*** | 104 |
|  |  | 15 | 37.85% | 12.87% | 44.66% | 14.34% | -2.608* | 104 |
|  |  | 10 | 38.30% | 13.05% | 47.73% | 13.33% | -3.678*** | 104 |
|  |  | 5 | 41.20% | 14.53% | 48.60% | 13.54% | -2.709** | 104 |
|  |  | 0 | 54.45% | 13.98% | 59.37% | 16.85% | -1.638 | 104 |
|  |  | -5 | 57.35% | 18.91% | 61.54% | 22.53% | -1.041 | 104 |
|  |  | -10 | 63.63% | 23.96% | 39.40% | 35.16% | 4.13*** | 89.573 |

^*^: P<0.05, ^**^: P<0.01, ^***^: P<0.001
